# Doppler Lidar Based on Mode-Locked Semiconductor Lasers

**DOI:** 10.3390/mi16111239

**Published:** 2025-10-30

**Authors:** Yibing Chen, Mengxi Zhou, Wenxuan Ma, Zhenxing Sun, Yuechun Shi, Hui Zou, Yunshan Zhang

**Affiliations:** 1 College of Electronic and Optical Engineering and College of Flexible Electronics (Future Technology), Nanjing University of Posts and Telecommunications, Nanjing 210023, China; 1023020517@njupt.edu.cn (Y.C.); 1223024726@njupt.edu.cn (M.Z.); ioozma@163.com (W.M.);; 2 College of Engineering and Applied Sciences, Nanjing University, Nanjing 210023, China; sunzhenxing@nju.edu.cn; 3 Yongjiang Laboratory, Ningbo 315201, China; yuechun-shi@ylab.ac.cn

**Keywords:** Doppler lidar, velocity measurement, mode-locked semiconductor laser, velocity measurement error, velocity resolution

## Abstract

This paper presents a Doppler lidar system based on a mode-locked semiconductor laser (ML-SL) source. The ML-SL consists of two sections: a Fabry–Pérot (F-P) cavity and a saturable absorber (SA) region. The system utilizes multiple phase-correlated modes of the optical frequency comb to acquire multiple Doppler shift signals; through cross-referencing of these signals, the robustness of the velocimetry system is enhanced. Experimental validation of precise velocity measurements for moving objects was conducted within the speed range of 0.005 m/s to 0.5 m/s. For target speeds of 0.563 m/s and 0.00563 m/s, the maximum and minimum absolute errors were 0.00064 m/s and 0.00003 m/s, respectively, with relative errors consistently below 1%. Comparative experiments demonstrated that utilizing multiple comb teeth reduces the maximum absolute error from 0.001286 m/s (observed when using a single tooth) to 0.000833 m/s. Furthermore, the velocity resolution of the system was analyzed: a frequency resolution of 30 Hz corresponds to a velocity resolution of 0.1117 m/s, while improving the frequency resolution to 1 Hz yields a velocity resolution of 0.0037 m/s.

## 1. Introduction

Lidar is a radar system capable of quickly acquiring information such as speed, distance and shape of target objects. Compared with traditional microwave radar, Lidar offers higher measurement accuracy and stronger anti-interference capability, thus finding wide application in the automotive industry [1], environmental monitoring [2], atmospheric monitoring [3], and other fields.

Doppler Lidar is an effective tool for measuring object speed, featuring non-contact operation, high resolution, and strong anti-interference capability. However, traditional Doppler Lidar typically employs a single-frequency light source, which suffers from shortcomings such as DC drift and insufficient anti-interference capability, while also imposing high requirements on laser linewidth [4,5]. To address the limitations of single-frequency light sources, a dual-frequency laser source scheme has been proposed [6,7,8]. Dual-frequency Doppler Lidar measures speed by detecting the Doppler frequency shift difference generated by two phase-locked laser wavelengths. This method converts the high requirement for laser linewidth into a requirement for microwave signal stability, effectively enhancing the system’s anti-interference capability and reducing the demands on laser linewidth. Consequently, obtaining phase-correlated dual-frequency lasers has become a key prerequisite. Existing methods for generating phase-correlated dual-frequency lasers include: utilizing the side-mode injection-locking characteristics of injection-locked semiconductor lasers [9]; generating four-wave mixing based on microwave modulation [10]; employing electro-optic modulators to produce carrier-suppressed modulation sidebands [11]; and filtering and selecting two specific modes from mode-locked lasers [12]. To date, differential Doppler frequency measurement primarily relies on dual wavelengths, even when mode-locked lasers are used [13]. Typically, optically injected semiconductor lasers and mode-locked lasers consist of numerous discrete components, resulting in complex system structures and high costs [12,14]—a factor that limits the widespread application of Doppler Lidar systems to some extent. Against this backdrop, developing a Doppler Lidar system based on a light source with a simple structure and easy integration holds significant research value.

With the rapid advancement of optical frequency comb technology, its outstanding time-frequency characteristics—such as broad spectral coverage, narrow pulse width, and high repetition rate stability—have been widely applied in precision spectroscopic measurements, time-frequency metrology, and absolute distance ranging. In particular, optical frequency combs based on mode-locked lasers have demonstrated exceptional performance in distance measurement, offering high precision, long range, and high-speed capabilities. The primary ranging methods include time-of-flight [15], interferometry [16], and hybrid schemes [17] that combine both approaches.

This paper proposes a Doppler lidar system based on a mode-locked semiconductor laser light source. The mode-locked semiconductor laser is composed of two segments: a Fabry–Perot (F-P) cavity and a saturable absorption (SA) region, featuring advantages such as low manufacturing cost, high stability, and simple driving (only requiring a DC power supply without complex input current devices). The multi-mode laser generated by injecting current into the F-P cavity can produce pulsed laser with a repetition frequency of 42 GHz and a linewidth of 60 kHz after being mode-locked by the saturable absorption region. The high repetition frequency of the semiconductor mode-locked laser helps reduce measurement errors, while its narrow linewidth is beneficial for improving velocity resolution. Experiments were conducted to measure the velocity of moving objects within a velocity range of approximately 0.005 m/s to 0.5 m/s. The relative error of the entire system is stable below 1%, with the maximum relative error being 0.5329%. The results indicate that this multi-wavelength scheme supports cross-reference measurement, improving velocity measurement accuracy to a certain extent. The system has the potential to measure objects with tiny velocity changes.

## 2. Methods

**Figure 1:**
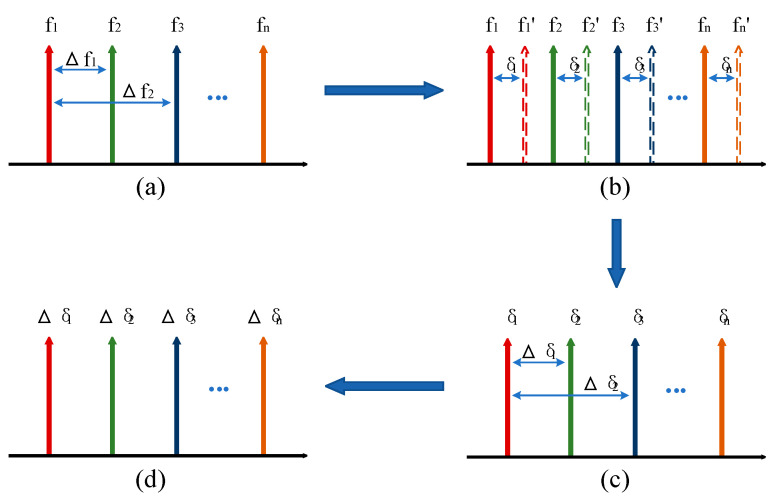
Principle of multi-wavelength differential Doppler velocimetry. (**a**) Optical frequency comb; (**b**) Reference signal and return signal. Among them, the solid arrow represents the reference signal, and the dashed arrow represents the return signal; (**c**) Doppler shift; (**d**) Differential Doppler shift. In the figure, $f_{n}$ represents different laser frequencies, $f_{n}^{\prime }$ denotes the different return laser frequencies after illuminating the moving object, $\delta_{n}$ indicates the Doppler shifts generated by different laser frequencies, and $\Delta \delta_{n}$ represents the differential Doppler shifts.

The principle of multi-wavelength differential Doppler velocimetry is illustrated in Figure 1. Its core concept lies in utilizing multiple discrete laser frequencies (corresponding to wavelengths $\lambda_{1},\lambda_{2}\ldots \lambda_{n}$ and frequencies $f_{1},f_{2}\ldots f_{n}$, as shown in Figure 1a) to simultaneously illuminate a moving object. The returned light carries the Doppler shifts associated with the object’s velocity, causing each frequency to shift to $f_{1}^{\prime },f_{2}^{\prime }\ldots f_{n}^{\prime }$ (as shown in Figure 1b). The Doppler shift for each frequency $\delta_{n}$ (where $f_{n}^{\prime }=f_{n}+\delta_{n}$) is related to the velocity v by Equation [13]:

(1) $$\delta_{n}=\frac{2vf_{n}}{c}$$

By solving the difference between the Doppler shifts corresponding (i.e., $\delta_{1}-\delta_{2}$) to any two frequencies (e.g., $f_{1}$ and $f_{2}$), the velocity can be accurately determined. This method effectively eliminates common-mode noise and improves measurement accuracy. The velocity of the moving target is

(2) $$v=\frac{c\Delta \delta_{n}}{2\Delta f_{n}}$$

where v is the velocity of the object, c is the speed of light in vacuum, $\Delta f_{n}$ is the beat frequency of two different frequencies (as shown in Figure 1a), and $\Delta \delta_{n}$ is the differential Doppler shift (as shown in Figure 1d) [10].

**Figure 2:**
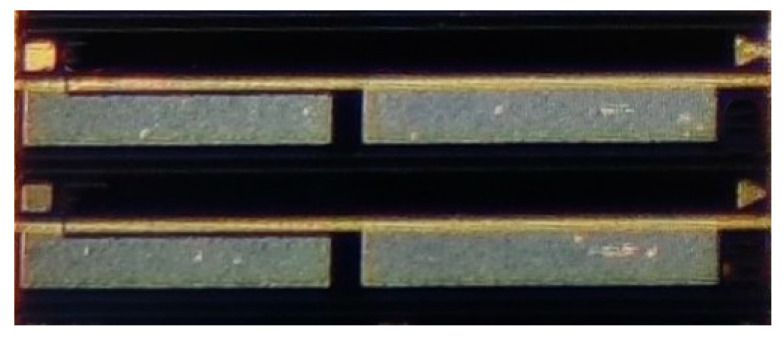
Chip diagram of the mode-locked semiconductor laser.

**Figure 3:**
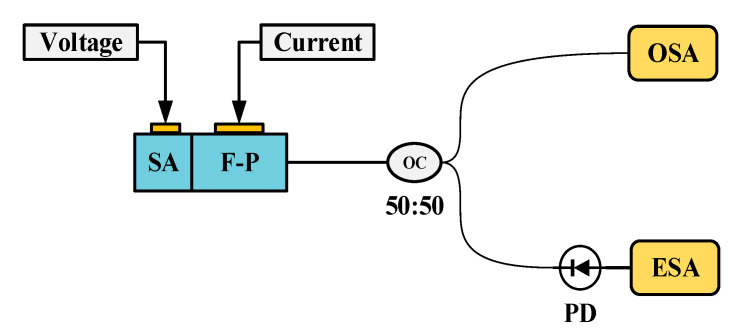
Testing system of the mode-locked semiconductor laser. OSA: Optical spectrum analyzer; ESA: Electronic spectrum analyzer; PD: Photodetector.

**Figure 4:**
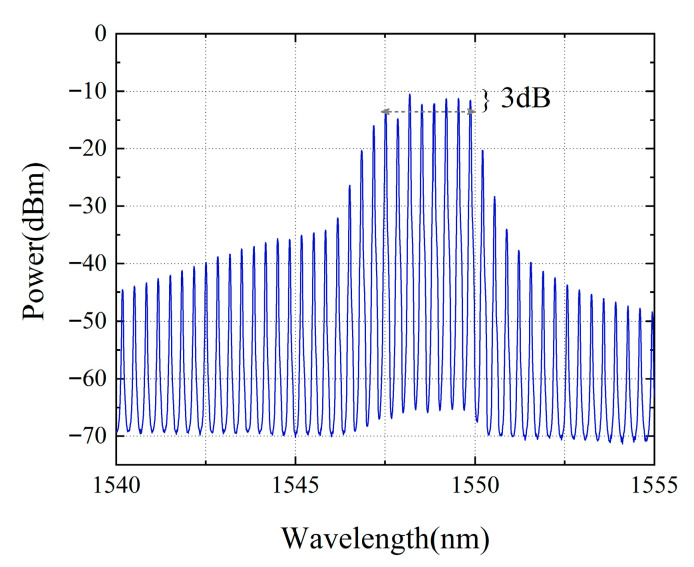
Spectrum of the mode-locked semiconductor laser.

**Figure 5:**
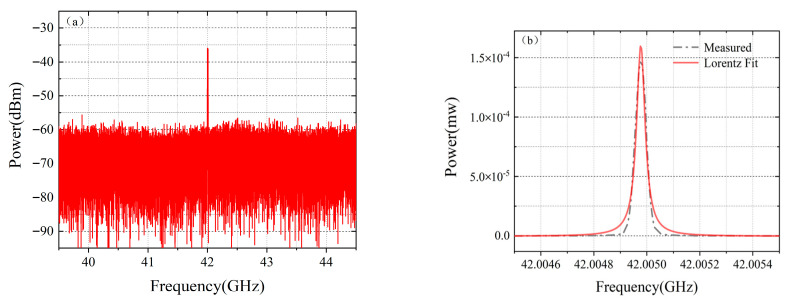
(**a**) Optical frequency comb beat note spectrum; (**b**) Lorentzian fit to the optical frequency comb beat note spectrum.

The velocity measurement system employs a two-segment mode-locked semiconductor laser as the light source. The laser chip(fabricated by ourself) is shown in Figure 2, with a total length of 1000 μm, consisting of two functional regions. The front segment is a Fabry–Perot (F-P) resonant cavity, accounting for 97% of the laser’s length, which is mainly responsible for generating stable multi-mode laser output. The rear segment is a saturable absorption region (SA), occupying 3% of the total length of the laser, and is made of a semiconductor material with light intensity-sensitive characteristics: it exhibits low absorption when the incident light intensity is high, while showing strong absorption under low-light conditions. There is an electrical isolation region between the two functional regions to ensure the independence of their respective working states. The laser operates at a central wavelength of 1550 nm, and its operating temperature is precisely controlled at 25 °C ± 0.1 °C by a thermoelectric cooler (TEC). Figure 3 shows the testing system of the mode-locked semiconductor laser. The output light of the laser passes through a 50:50 beam splitter, one beam of which is connected to a spectrometer (YOKOGAWA AQ6370D Optical spectrum Analyzer, Tokyo, Japan), and the other beam is connected to a spectrum analyzer (Keysight EXA Singnal Analyzer N9010B, Houston, TX, USA) through a photodetector (PD)(LPTBR-200, Suzhou, China). Under the operating conditions where a driving current of 50 mA is injected into the F-P cavity and a reverse bias voltage of −0.5 V is applied to the SA region, the laser can generate 6 stable modes with balanced power (the power of each mode differs from the main mode by no more than 3 dB), as shown in Figure 4. Figure 5 demonstrates the spectral characteristics of the mode-locked semiconductor laser: (a) is optical frequency comb beat note spectrum of 42 GHz. To extract the full width at half maximum (FWHM) linewidth of the beat note spectrum, we fitted its spectral profile with a Lorentzian function. The resulting fitted curve is shown as Figure 5b. The linewidth value obtained from the fitting result is 60 kHz. This performance provides an important guarantee for the system to achieve high-precision velocity measurement.

**Figure 6:**
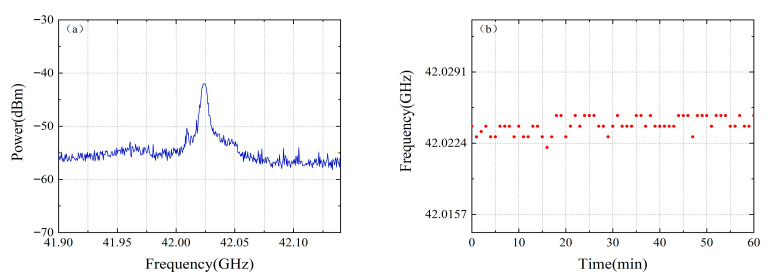
(**a**) Peak frequency stability measurement of the mode-locked laser beat note. (**b**) Long-term frequency stability test of the optical frequency comb’s beat signal (1 h). The blue curve represents the continuous one-hour monitoring of the maximum power of the beat signal from the mode-locked laser. The red dots indicate the beat frequency of the mode-locked laser recorded at one-minute intervals over the one-hour monitoring period.

The stability of the mode-locked laser’s beat signal was evaluated over a one-hour period, as shown in Figure 6. The recorded data revealed a maximum peak frequency jitter of 2 MHz. Attributable to this jitter being four orders of magnitude smaller than the 42 GHz beat frequency, its influence on the measurement precision is negligible.

## 3. Results and Discussion

### 3.1. Experimental Setup

**Figure 7:**
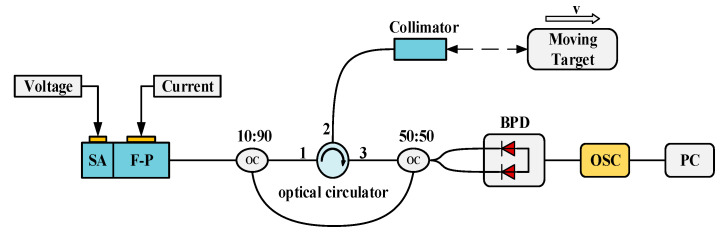
Experimental system for Doppler velocity measurement based on a semiconductor mode-locked laser. F-P: Fabry–Perot cavity; SA: Saturable absorption region; OC: Optical coupler; CIR: Circulator; BPD: Balanced photodetector; OSC: Real-time oscilloscope; PC: Personal computer.

Figure 7 shows the experimental system for Doppler velocity measurement based on a semiconductor mode-locked laser. The pulsed light output by the mode-locked laser is split into two paths by a 10:90 optical beam splitter: 10% of the light serves as the reference light and is input into the coupler; the remaining 90% acts as the measuring light and is input into port 1 of the optical circulator. The measuring light exits from port 2 of the optical circulator, is collimated by a collimator, and then irradiates the moving object. The light reflected by the object is collected by the collimator, transmitted back to port 2 of the optical circulator, and output through its port 3. Subsequently, the returned measuring light and the reference light are coupled via a 2 × 2 optical coupler. The coupled optical signal is input into a balanced photodetector (BPD) for detection. The detected electrical signal is collected by an oscilloscope, and finally, the collected data is processed at the PC end to extract the Doppler frequency shift information, based on which the corresponding velocity is calculated.

### 3.2. Experimental Results

**Figure 8:**
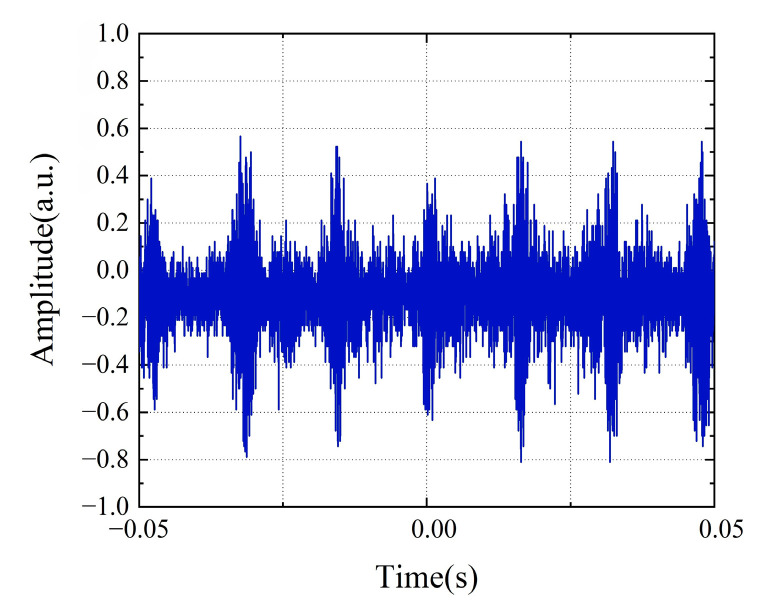
Time-domain waveform collected by the real-time oscilloscope (OSC).

**Figure 9:**
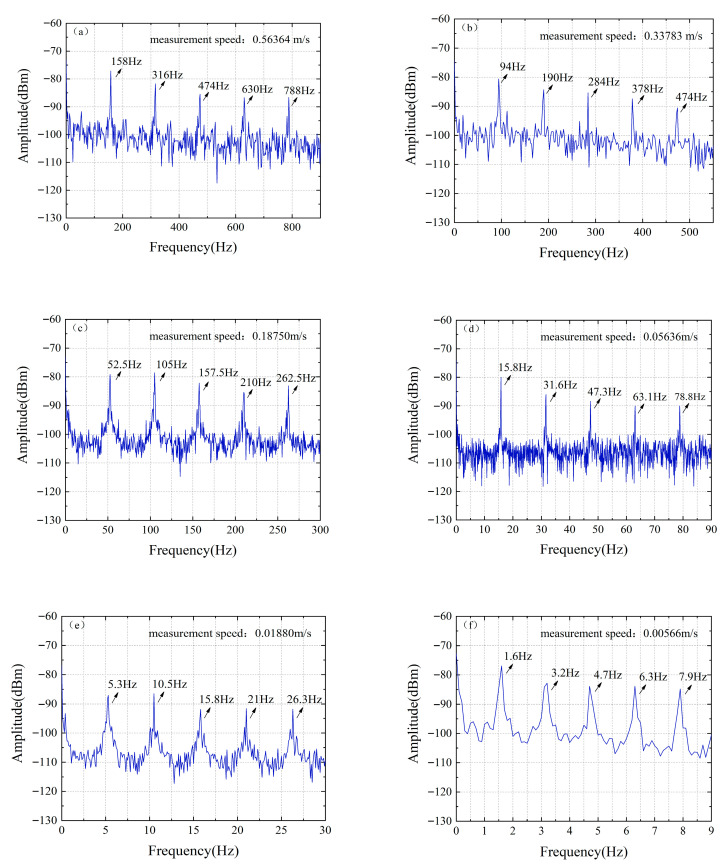
Differential Doppler shifts at different set speeds obtained by FFT: (**a**) Set speed: 0.563 m/s; (**b**) Set speed: 0.337 m/s; (**c**) Set speed: 0.187 m/s; (**d**) Set speed: 0.0563 m/s; (**e**) Set speed: 0.0187 m/s; (**f**) Set speed: 0.00563 m/s.

**Figure 10:**
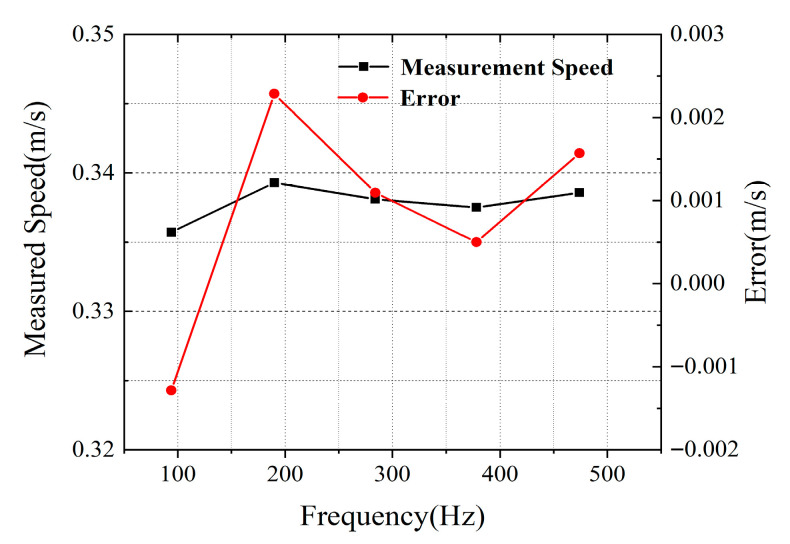
Measurement results and error analysis of different comb teeth in the optical frequency comb when the target speed is 0.0337 m/s. Measurement error is an important factor affecting the speed measurement accuracy.

The object was placed at a distance of 1 m from the collimator. Figure 8 shows the typical time-domain waveform characteristics acquired by a real-time oscilloscope (OSC). Different from the sinusoidal waveform generated by the traditional continuous-wave Doppler velocity measurement system, the time-domain waveform of this system presents discontinuous characteristics with a pulse base due to the inclusion of multiple beat frequency components in the measurement signal, and its signal envelope contains the Doppler frequency shift information to be measured. By squaring the time-domain signal and then performing Fourier transform, the differential Doppler frequency shift of the system can be extracted, as shown in Figure 9. In the experiment, six speed points of 0.563 m/s, 0.337 m/s, 0.187 m/s, 0.0563 m/s, 0.0187 m/s, and 0.00563 m/s were selected for measurement. The results show that the six main modes of the mode-locked semiconductor laser produce five clear differential Doppler frequency components. Taking the measured speed of 0.337 m/s as an example, the system detected five characteristic frequencies of 94 Hz, 190 Hz, 284 Hz, 378 Hz, and 474 Hz, which correspond to the differential Doppler frequency shifts generated by the fundamental wave and harmonic components. This multi-frequency measurement mechanism realizes the cross-validation of speed information and significantly improves the measurement robustness of the system. Figure 10 shows in detail the measurement results and error analysis of different comb teeth in the optical frequency comb when the target speed is 0.337 m/s. The experimental data show that the average measured speed calculated based on different comb teeth is 0.33783 m/s, with an absolute error of 0.00083 m/s and a relative error of only 0.2473%. This result verifies that the system can still maintain high measurement accuracy in low-speed measurement. 

**Figure 11:**
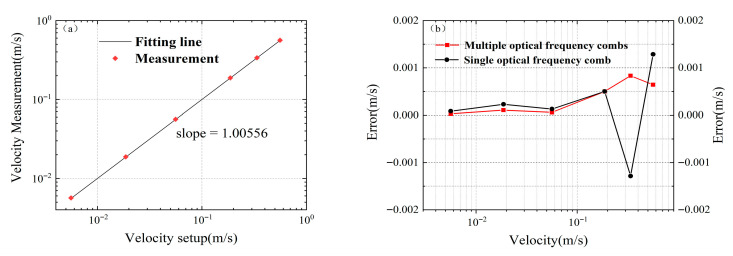
(**a**) Fitting curve of set speed and measured speed; (**b**) Measurement errors of single comb tooth speed measurement and multiple comb teeth speed measurement.

In multi-wavelength velocity measurement technology, the measurement error is closely related to the frequency interval between different wavelengths: the larger the frequency interval, the smaller the measurement error. The mode-locked semiconductor laser adopted in this system has a repetition frequency of 42 GHz, which enables the system to achieve extremely high velocity measurement accuracy. Figure 11a shows the comparison results between the measured speed and the set speed, presenting the linear fitting of the set speed and the measured speed. Theoretically, the closer the slope of the fitting curve is to 1, the higher the accuracy of the measured speed. The slope of the straight line in the figure is 1.00556, which indicates that the deviation of the measured speed by the system is very small. Figure 11b shows the measurement errors of single comb tooth velocity measurement and multiple comb teeth velocity measurement. It can be observed that multiple comb teeth velocity measurement reduces the maximum error from 0.001286 m/s to 0.000833 m/s. In multiple comb teeth velocity measurement, for the set speeds of 0.563 m/s and 0.00563 m/s, the absolute values of the maximum and minimum measurement errors are 0.00064 m/s and 0.00003 m/s, respectively, with corresponding relative errors of 0.1137% and 0.5329%.The results show that the entire system has high velocity measurement accuracy within the measurement range, and the method of multiple comb teeth velocity measurement can improve the robustness of velocity measurement.

**Figure 12:**
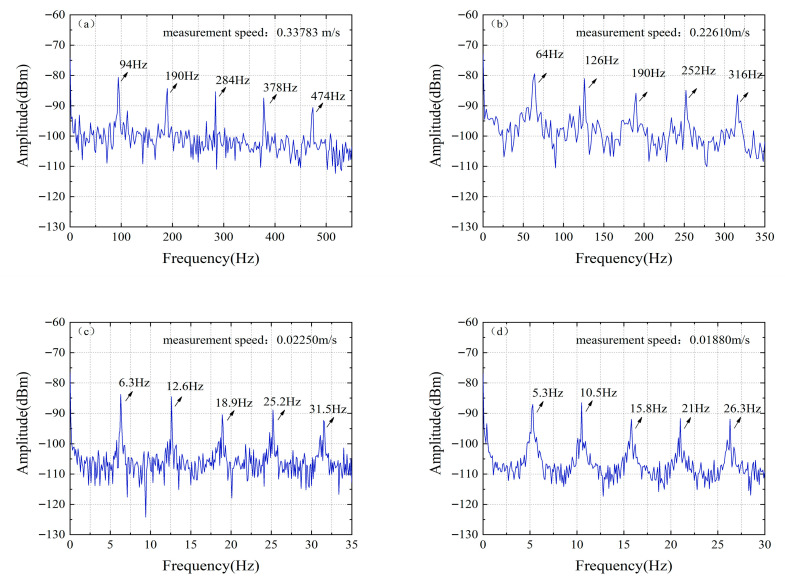
Resolution of the velocity measurement system with different FFT frequency resolutions. (**a**,**b**) Velocity resolution: 0.1117 m/s, Frequency resolution: 30 Hz; (**c**,**d**) Velocity resolution: 0.0037 m/s, Frequency resolution: 1 Hz.

Velocity measurement resolution is another key indicator for evaluating the performance of a velocity measurement system, which is mainly constrained by both the linewidth characteristics of the mode-locked semiconductor laser and the frequency resolution of the Fast Fourier Transform (FFT). In this system, since the beat frequency signal of the adopted mode-locked semiconductor laser has an excellent narrow linewidth characteristic of approximately 60 kHz, the velocity measurement resolution of the system mainly depends on the frequency resolution of the FFT algorithm. The FFT frequency resolution is directly determined by the time length of the waveform data collected by the oscilloscope, and this parameter has a decisive impact on the final velocity measurement accuracy. Figure 12 shows the velocity resolution of the velocity measurement system with different FFT frequency resolutions. When the frequency resolution is 30 Hz, the velocity resolution between Figure 12a,b is 0.1117 m/s. When the frequency resolution is 1 Hz, the velocity resolution of Figure 12c,d is 0.0037 m/s.

**Figure 13:**
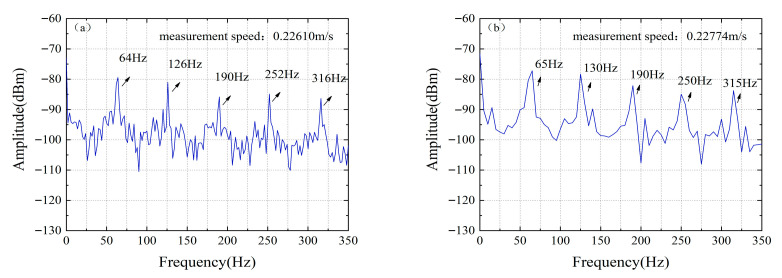
Effect of FFT Frequency Resolution on Measurement Error. (**a**) 2 Hz frequency resolution. (**b**) 5 Hz frequency resolution.

As shown in Figure 13, increasing the FFT frequency resolution effectively reduces velocimetry error. In a test at 0.226 m/s, optimizing the resolution from 5 Hz to 2 Hz reduced the measurement error by an order of magnitude (from 0.00174 m/s to 0.0001 m/s), validating the critical role of high frequency resolution in enhancing measurement accuracy.

The results indicate that by collecting waveform data with a longer time length via the oscilloscope, the frequency resolution of FFT can be improved, thereby further enhancing the velocity resolution.

## 4. Discussion

The theoretical limits of multi-wavelength velocimetry are governed by the frequency resolution of the acquisition equipment (as referenced in relevant studies) for the lower bound and the bandwidth of the balanced photodetector (BPD) for the upper bound.

(3) $$\delta_{n}=\frac{2vf_{n}}{c}$$

where $\delta_{n}$ denotes the Doppler shift, v is the velocity of the object, $f_{n}$ represents the frequency of the measurement light, and c is the speed of light.

According to the Doppler velocimetry principle, a higher target velocity generates a greater Doppler frequency. When this frequency exceeds the bandwidth of the balanced photodetector (BPD), the signal cannot be effectively detected, leading to measurement failure. Compared with single-frequency velocimetry, the multi-frequency technique significantly reduces the required detectable Doppler frequency through a differential processing scheme. Its core principle lies in utilizing the beat frequency signal generated by two lasers of different wavelengths to determine velocity. This beat frequency is substantially lower than the optical frequencies of the individual lasers. As can also be derived from the Doppler velocimetry formula, a lower optical frequency results in a smaller corresponding Doppler frequency. Therefore, multi-frequency velocimetry possesses an inherent advantage for high-speed target measurement, as it effectively relaxes the bandwidth requirements for the BPD.

In this detection system, the final-stage differential Doppler frequency must not exceed the bandwidth limit of the BPD. The BPD used in the experiment has a bandwidth of 250 MHz, and there are five stages of differential Doppler frequencies in total. Calculations show that the theoretical maximum measurable velocity of the system is 1.7857 × 10^5^ m/s.

**Figure 14:**
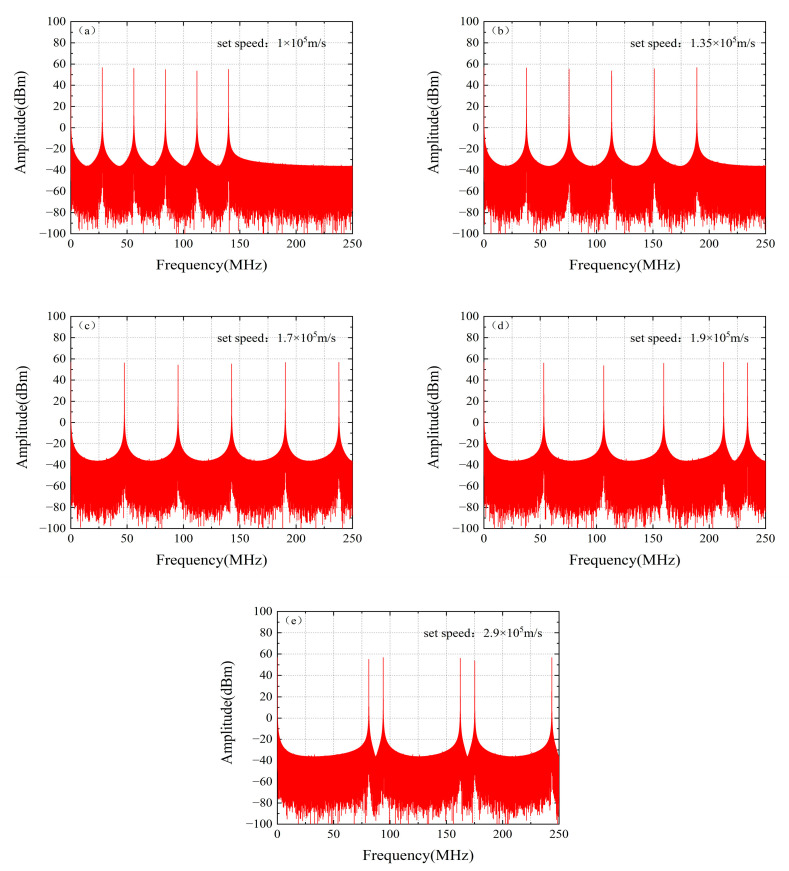
Differential Doppler shifts at different set speeds obtained by FFT: (**a**) Set speed: 1 × 10^5^ m/s; (**b**) Set speed: 1.35 × 10^5^ m/s; (**c**) Set speed: 1.7 × 10^5^ m/s; (**d**) Set speed: 1.9 × 10^5^ m/s; (**e**) Set speed: 2.9 × 10^5^ m/s.

To validate the aforementioned theory, we conducted corresponding simulation analyses. In the simulations, the optical frequency comb was configured with a frequency spacing of 42 GHz, a carrier wavelength of 1550 nm, and uniform comb tooth amplitudes of 1. For five distinct target velocities—1 × 10^5^ m/s, 1.35 × 10^5^ m/s, 1.7 × 10^5^ m/s, 1.9 × 10^5^ m/s, and 2.9 × 10^5^ m/s—the corresponding Doppler frequency shift spectra were simulated and extracted, with the results shown in Figure 14.

For the cases of 1 × 10^5^ m/s, 1.35 × 10^5^ m/s, and 1.7 × 10^5^ m/s, the generated Doppler frequency shifts all fall within the bandwidth of the BPD. Complete Doppler frequency components can be clearly identified in Figure 14a–c, with each component accurately corresponding to the theoretical velocity. However, in Figure 14d,e, frequency aliasing occurs in the spectra corresponding to velocities of 1.9 × 10^5^ m/s and 2.9 × 10^5^ m/s. This is because some high-frequency components in the signal exceed the Nyquist frequency and are erroneously identified as low-frequency components during the sampling process, making it impossible to accurately extract the Doppler frequency and consequently preventing correct determination of the target velocity.

## 5. Conclusions

This study successfully demonstrates a Doppler velocity measurement system based on a mode-locked semiconductor laser. The scheme uses a mode-locked semiconductor laser as the light source, which solves the problem of laser multi-mode correlation in measurement at a relatively low cost, realizes multi-wavelength collaborative velocity measurement, and effectively improves the velocity measurement accuracy. Experiments show that by using the cross-reference of multiple comb tooth modes, the maximum absolute error can be reduced from 0.001286 m/s in the case of a single comb tooth mode to 0.000833 m/s. Within the speed range of 0.005 m/s to 0.5 m/s, the system measurement error is lower than 1% of the target speed value (the maximum relative error is 0.5329%). The speed resolution of the system can be further improved by increasing the oscilloscope acquisition time to enhance the frequency resolution (as mentioned earlier, a frequency resolution of 1 Hz corresponds to a speed resolution of approximately 0.0037 m/s), and the experimental data verify the effectiveness of this method.

In summary, the ML-SL velocity measurement scheme proposed in this work provides a new technical solution for Doppler velocity measurement due to its compact structure, low cost, simple driving, and high accuracy.

## Data Availability

This data is unavailable for confidentiality purposes.

## References

[1] Fackelmeier A., Morhart C., Biebl E. (2008). Dual frequency methods for identifying hidden targets in road traffic. Advanced Microsystems for Automotive Applications 2008.

[2] Rydhmer K., Strand A. (2016). Applied Hyperspectral LIDAR for Monitoring Fauna Dispersal in Aquatic Environments. Master’s Thesis.

[3] Vaughan J. (1998). Coherent laser spectroscopy and Doppler lidar sensing in the atmosphere. Phys. Scr..

[4] Mocker H.W., Bjork P.E. (1989). High accuracy laser Doppler velocim eter using stable long-wavelength semiconductor lasers. Appl. Opt..

[5] Sharma U., Chen G., Kang U. (2005). Fiber optic confocal laser Doppler velocimeter using an all-fiber laser source for high resolution measurements. Opt. Express.

[6] Morvan L. (2002). Building blocks for a two-frequency laser lidar-radar: A preliminary study. Appl. Opt..

[7] Shangguan M., Xia H., Wang C., Qiu J., Lin S., Dou X., Pan J.W. (2017). Dual-frequency Doppler lidar for wind detection with a superconducting nanowire single-photon detector. Opt. Lett..

[8] Cheng C., Lee C., Lin T., Lin F. (2012). Dual-frequency laser Doppler velocimeter for speckle noise reduction and coherence enhancement. Opt. Express.

[9] Lu Z., Zhang Y., Zeng J. (2019). High Precision Dual Frequency Doppler Lidar Based on Monolithic Integrated Two-Section DFB Lasers. IEEE Photonics J..

[10] Chen C., Lu D., Guo L., Zhao W., Wang H., Zhao L. Dual-frequency Laser Doppler Velocimeter based on Integrated Dual-mode Amplified Feedback Laser. Proceedings of the ACP.

[11] Wang L., Zhao L., Zhang Y., Wu Y., Xia H. (2023). Tunable dual-frequ ency coherent Doppler lidar using bi-directional electro-optic modula tion in a Sagnac loop. Opt. Commun..

[12] Vercesi V., Onori D. (2015). Frequency-agile dual-frequency lidar for integrated coherent radar-lidar architectures. Opt. Lett..

[13] Song G., Lu D., Zhang Z., Guo F., Zhou D., Zhao L. Differential Doppler Velocity Measurement Using a Distributed Bragg Reflector Mode-Locked Laser. Proceedings of the ACP/POEM.

[14] Cheng C.H., Lin L.C., Lin F.Y. (2014). Self-mixing dual-frequency laser Doppler velocimeter. Opt. Express.

[15] Minoshima K., Matsumoto H. (2000). High-accuracy measurement of 240-m distance in an optical tunnel by use of a compact femtosecond laser. Appl. Opt..

[16] Schuhler N., Salvadé Y., Lévêque S., Dändliker R. (2006). Frequency-comb-referenced two-wavelength source for absolute distance measurement. Opt. Lett..

[17] Coddington I., Swann W., Nenadovic L. (2009). Rapid and precise absolute distance measurements at long range. Nat. Photon..

